# Comparative Effect of Crude and Commercial Enzyme on the Juice Recovery from Bael Fruit (*Aegle marmelos* Correa) Using Principal Component Analysis

**DOI:** 10.1155/2013/239839

**Published:** 2013-03-06

**Authors:** Anurag Singh, H. K. Sharma, Sanjay Kumar, Ashutosh Upadhyay, K. P. Mishra

**Affiliations:** ^1^Department of Food Technology, FET, RBS Engineering Technical Campus, Bichpuri, Agra 283105, India; ^2^Food Engineering and Technology Department, Sant Longowal Institute of Engineering and Technology (SLIET), Longowal, Sangrur, Punjab 148106, India; ^3^National Institute of Food Technology Entrepreneurship and Management, Kundli, Sonipat, Haryana 131028, India; ^4^Faculty of Engineering and Technology, Mahatma Gandhi Chitrakoot Gramodaya Vishwavidyalaya, Chitrakoot, Satna, Madhya Pradesh 485331, India

## Abstract

The effect of incubation time, incubation temperature, and crude enzyme concentration was observed on the yield, viscosity, and clarity of the juice obtained from bael fruit pulp. The recommended enzymatic treatment conditions from the study were incubation time 475 min, incubation temperature 45°C, and crude enzyme concentration 0.20 mL/25 g bael fruit pulp. The recovery, viscosity, and clarity of the juice under these conditions were 82.9%, 1.41 cps, and 21.32%T, respectively. The variables, clarity, and yield were found as principal components for comparing different samples of the juice treated with enzyme.

## 1. Introduction 

The bael fruit (*Aegle Marmelos *Correa) has been attributed with various nutritional and therapeutic properties. The fruit has excellent aroma which is not destroyed even during processing [1]. The bael fruit pulp contains many functional and bioactive compounds such as carotenoids, phenolics, alkaloids, coumarins, flavonoids, terpenoids, and other antioxidants which may protect against chronic diseases [2]. It has been surmised that the psoralen in the pulp increases tolerance of sunlight and aids in the maintaining of normal skin color and is considered fruitful in the treatment of leucoderma. The marmelosin (C_13_H_12_O_3_) content, found in the bael fruit, is considered as panacea of various stomach ailments [3]. Bael fruit, because of its hard shell, mucilaginous texture, and numerous seeds, is not popular as fresh fruit. However, the excellent flavor and nutritive and therapeutic value of bael fruits show potential for processing into values added products. Bael is commercially considered as an important fruit, but the potential of the fruit is not fully tapped.

The edible pulp, 100 g of bael fruit contains 61.5 g water, 1.8 g protein, 0.39 g fat, 1.7 g minerals, 31.8 g carbohydrate, 55 mg carotene, 0.13 mg thiamine, 1.19 mg riboflavin, 1.1 mg niacin, and 7 to 21 mg ascorbic acid [4]. Generally, three methods of juice extraction are employed, namely, cold, hot, and enzymatic methods. The use of fungal enzyme in fruit juice extraction had shown significant increase in juice recovery as compared to cold and hot extraction methods. The enzymes, mainly pectinases, and cellulases assist in pectin and cellulolytic hydrolysis, respectively, which cause a reduction in pulp viscosity and a significant increase in juice yield [5]. 

The extraction of bael juice on large scale has not been explored for its commercial scale viability and exploitation but conventionally, the extraction includes addition of water to pulp, boiling and pressing of juice from the mixture. The residual pulp remaining after juice extraction still contains valuable extractable material such as particulate, flavour, and soluble solids which may further improve the final quality of the juice. By adding cell wall liquefying enzymes, it is possible to further extract valuable juice components from pulp.

The area requires wider research in terms of utilization of residue, enhanced juice yield with optimum overall acceptability. The application of commercial enzyme for the different juice clarification is reported by several researchers [6–8]. It is more economical to use crude enzyme (spore free and produced from GRAS fermentation) for the improvement of juice yield and clarity. Therefore, the present study was undertaken to use crude enzyme from *A. Niger* for the treatment of the bael pulp to improve the juice yield with optimum overall acceptability and examine the comparative effect of enzymes in crude and purified form by using Principal Component Analysis (PCA).

## 2. Materials and Methods

### 2.1. Materials

Fully ripe fresh bael fruits (*Aegle marmelos* Correa) of Kagazi variety, without any visual defects, were procured from Agricultural farm of RBS College, Bichpuri, Agra (India). The bael fruits were broken by hammering, and the pulp was scooped out with the help of stainless steel spoon. The scooped pulp was homogenized by blending manually. This pulp was used to extract juice.

### 2.2. Crude Enzyme Preparation

The strain of *Aspergillus niger* NCIM 548 was obtained from the National Chemical Laboratory, Pune. The strain was cultured on potato dextrose agar slant and subcultured after every 6–8 weeks. This strain was used for the production of crude enzyme under solid state fermentation (SSF) using wheat bran, corn bran, and kinnow peel (in 2 : 1 : 2 ratio) as substrate as per the method reported by Kumar et al. [9]. The enzyme contained 50 U/mL of the pectinase and 20 U/mL of the cellulase and was used for the treatment of bael fruit pulp to improve the juice yield and quality.

### 2.3. Experimental Design and Statistical Analysis

Response surface methodology (RSM) was used in designing the experiments as it provides the modeling and analysis of the problem in which several variables influence the output parameter, and the objective is to optimize this parameter [10]. RSM provides a reduced number of experimental runs needed to obtain sufficient information for statistically acceptable results. A five-level three-factor central composite rotatable design was employed. The independent variables were the temperature of enzyme treatment (*X*_1_), time of treatment (*X*_2_), and used enzyme concentration (*X*_3_). The variables and their levels were chosen based on the limited literature available on enzymatic hydrolysis of fruits [6–8]. These were the temperature (*X*_1_; 35–55°C), time (*X*_2_; 210–540 min) of the enzymatic treatment, and concentration of enzyme (*X*_3_; 0.06–0.20 mL/25 g pulp). The experimental design matrix in coded (*x*) form and at the actual level (*X*) of variables is given in Table 1. A total of 20 experiments were carried out by using crude enzyme under different experimental conditions as given in Table 2. The response function (*Y*) was related to the coded variables by a second degree polynomial equation (1) as follows:

(1) y=b0+b1x1+b2x2+b3x3  +b12x1x2+b13x1x3+b23x2x3+b11x21+b22x22+b33x23+ε.  

The coefficients of the polynomial were represented by *b*_0_ (constant), *b*_1_, *b*_2_, *b*_3_ (linear effects), *b*_12_, *b*_13_, *b*_23_ (interaction effects), *b*_11_, *b*_22_, *b*_33_ (quadratic effects), and *ε* (random error). 

### 2.4. Commercial Enzyme Treatment of Bael Pulp under Optimized Conditions

The pretreatment conditions based on the application of commercial enzyme on the juice recovery from bael fruit were also optimized in our laboratory using CCRD design of Response Surface Methodology. The optimum conditions observed were concentration of pectinase 5 mg/25 g of pulp (1.64 IU/mg), time 425 min, and temperature 47°C as given in Table 4 [7].

### 2.5. Analysis of Response Variables

#### 2.5.1. Enzymatic Treatment and Juice Yield

The bael pulp for the treatment of enzymes was prepared as per the procedure adopted by Singh et al. [7]. The temperature was adjusted to the required level by using a high precision water bath (Seco, Model 129, India) for all enzymatic treatment combinations. At the completion of the enzyme treatment, the treated mixture was filtered by using 6-fold cheese cloth, and the extract was heated at 90°C for 5 min to inactivate the enzyme. The extract thus obtained was considered as clear juice for determining the juice yield. 

#### 2.5.2. Clarity and Viscosity

The clarity was determined by the method given by Krop and Pilnik [11], and viscosity of the juice was determined as per the method reported by Ranganna [12]. The juice was shaken, and 10 mL portion of juice was centrifuged at 3000 rpm for 10 min to remove pulp coarse cloud particles. The clarity of the juice obtained was determined by measuring the transmittance at a wavelength of 590 nm using UV-VIS spectrophotometer (UV 5704SS, Electronics Corporation of India Ltd.). 

Time required to flow through the capillary section of the Oswald viscometer was noted using a stopwatch for the reference and the sample at 20 ± 2°C.

### 2.6. Optimization and Verification 

The optimal level of three independent variables, namely, temperature (*X*_1_), time (*X*_2_) of the enzymatic treatment, and concentration of enzyme (*X*_3_), was established with the help of graphical and numerical optimization procedures resulting to desirable responses which were maximum yield, maximum clarity, and minimum viscosity. For graphical optimization, a three-dimensional response surface was plotted by varying the two variables in the experimental range while keeping rest one variable constant at the centre point. The exact optimum value of individual and multiple responses was determined by a numerical optimization process using Design Expert software (DE–6) (Trial version; STAT-EASE Inc., Minneapolis, MN, USA). To verify the predicted results, the experiments were conducted at the optimized conditions. The predictive models were validated on the basis of *R* ^2^, adjusted *R* ^2^, *F*-value, Lack of fit, and so forth obtained from the analysis of the experimental data by using Design Expert software.

### 2.7. Comparative Study Using Principal Component Analysis (PCA)

For exploring the feasibility of the application of the crude enzyme in place of commercial enzymes, the usages of commercial enzymes were compared with the crude enzymes under the optimized conditions, on the same variety of bael fruit. Principal component analysis was carried out by using software Minitab version 16.1 (Trial version; Minitab Inc., USA) to form a smaller number of uncorrelated variables from a large set of data. A large number of variables are reduced to a few variables called principal components (PCs) that describe the greatest variance in the data analyzed [13]. The technique helps to understand the similarities and differences between the samples treated with crude and commercial enzymes separately and also establishes an interrelationship between the measured properties of the samples.

## 3. Results and Discussion

### 3.1. Optimization of Pretreatment Conditions by Using Crude Enzyme

The juice extracted from enzyme treated and untreated (control) pulp was evaluated for the juice yield (%), viscosity, and clarity. Table 2 shows the juice yield, apparent viscosity, and clarity under the different experimental conditions by using crude enzyme. It is clear from the data obtained that the juice has been improved significantly with respect to quantity as well as quality by the enzymatic treatment.

#### 3.1.1. Adequacy of the Models for the Different Responses

The model was judged for its fitness and adequacy by the coefficient of determination (*R* ^2^), which is the ratio of the explained variation to the total variation. The closer the *R* ^2^ value to unity, the better the empirical model fits the actual data. The coefficients of determination, *R* ^2^, in the model were 0.9764, 0.9738, and 0.9806 (Table 3) for the regressed models predicting the juice yield, viscosity, and clarity, respectively, suggesting a good fit for the models. The adjusted *R* ^2^ was a corrected value for *R* ^2^ after elimination of the unnecessary model terms, which was very close to their corresponding *R* ^2^ values for all the responses. Higher values of adjusted *R* ^2^ also confirm the significance of the models.

The *F* value of 45.92, 41.38, and 56.04 for juice yield, viscosity, and juice clarity, respectively, also inferred that the models were significant (*P* < 0.001). The model for the juice yield, viscosity, and clarity can be derived by using the coefficients (Table 3) for the predictions of the data. The predicted models seemed to reasonably represent the observed values. Thus, the responses were adequately explained by the model.

The coefficient of variation (CV) describes to which extent the data are dispersed and is defined as a measure of residual variation of the data relative to the size of the mean; the small values of CV give better reproducibility. The small CV values 1.31, 1.33, and 1.34 (Table 3) of juice yield, viscosity, and clarity, respectively, suggested that the experimental results were precise and reliable. 

#### 3.1.2. Response Surface Analysis

(1)  *Juice Yield.* The juice yield ranged from 69.8% to 83.7% (Table 2). The yield was minimum when crude pectinase enzyme 0.06 mL/25 g pulp was used for 210 min at 55°C, whereas the maximum juice yield was at 0.25 mL/25 g crude pectinase enzyme concentration for 375 min at 45°C. 

**Figure 1:**
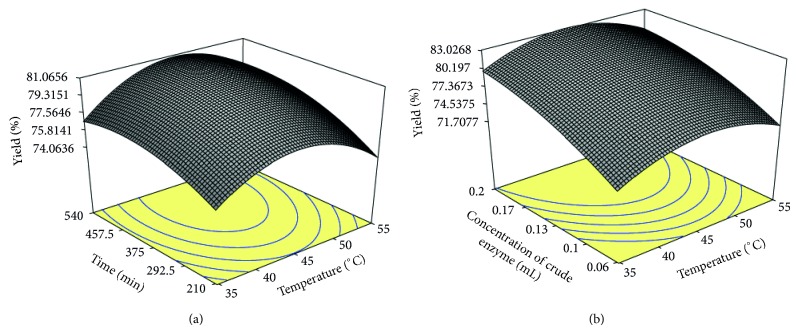
Response surface plots of juice yield as a function of (a) time and temperature and (b) concentration of crude pectinase enzyme and temperature.

To understand the interaction of different variables and to find the optimum level of each variable, the response surface curves were plotted. The response surface curves for juice yield are shown in Figures 1(a) and 1(b). Each response surface curve explains the effect of two variables while keeping the third variable at middle level.  Figure 1(a) represents the interactive effect of incubation temperature (*X*_1_) and incubation time (*X*_2_) on the juice yield, whereas the concentration of crude enzyme (*X*_3_) was kept at middle level, that is, 0.13 mL/25 g of bael pulp. The juice yield increased with the increase in both time and temperature up to 473 min of time and 45.8°C temperature. With further increase in temperature, the yield slightly decreased but remains unaffected of increase in incubation time. The decrease in juice yield with increasing temperature beyond 45.8°C may be due to denaturation of protein which may lead to decrease in enzyme activity at higher temperature. The results are supported by the findings of Kaur et al. [6], who reported that the maximum juice yield from guava is obtained by pectinolytic enzyme treatment of pulp at 43.3°C temperature for 447 min of time.

Figure 1(b) presents the interaction effect of incubation temperature (*X*_1_) and crude enzyme concentration (*X*_3_) to juice yield. At higher temperature and enzyme concentration, the juice yield followed a linear behaviour which reflects that with increase in enzyme concentration and temperature, juice yield increased up to maximum concentration of enzyme, that is, 0.20 mL/25 g pulp and 45°C temperature, respectively. Thereafter, the juice yield decreased slowly beyond 45°C, which may be due to decrease in enzyme activity at higher temperature. The increase in plum juice yield with pectinase enzyme is also supported by Singh and Das Gupta [14] who reported that pectinases increase the juice yield from 48% to 77.5%.

**Figure 2:**
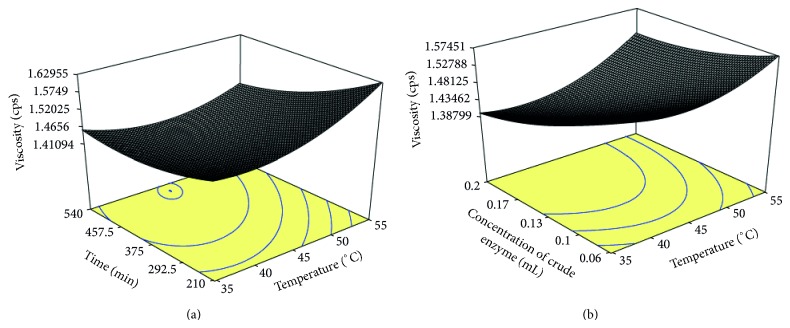
Response surface plots of viscosity of juice as a function of (a) time and temperature and (b) concentration of crude pectinase enzyme and temperature.

(2)  *Viscosity.* The viscosity of the bael juice ranged from 1.40 to 1.69 cps under the different experimental conditions (Table 2). The minimum viscosity was observed at 0.25 mL/25 g crude enzyme concentration for 375 min at 45°C. The corresponding condition for maximum viscosity was 0.06 mL/25 g crude enzyme concentration, for 210 min at 55°C. It is clear from Figure 2(a) that viscosity decreased with increase in both time and temperature up to 510 min of time and 44°C temperature. The findings are in accordance with Kumar and Sharma [8] who reported that the viscosity of the juice decreased with increase in temperature up to 47°C. The temperature increases the rate of enzymatic reactions. Upon enzyme treatment, degradation of pectin leads to a reduction of water holding capacity, and consequently free water was released to the system thus reducing the viscosity of the juice.

Figure 2(b) presents the interaction effect of incubation temperature (*X*_1_) and crude enzyme concentration (*X*_3_) to viscosity. The viscosity decreased with the increase in both concentration of enzyme and incubation temperature up to 0.20 mL/25 g of pulp of crude enzyme concentration and 40°C temperature. Lee et al. [15] observed that the viscosity of the juice decreases when the enzyme concentration is increased from 0.01% to its maximum value (0.1%). 

(3)  *Juice Clarity.* The clarity of the extracted juice was in a range from 16.60 to 21.10 %T. The minimum clarity was observed when 0.13 mL/25 g pulp, crude enzyme concentration was used for 375 min at 61.81°C. The rate of clarification increases with an increase in enzyme concentration may be due to the exposure of positively charged protein beneath, which reduces electrostatic repulsion between cloud particles causing these particles to aggregate into larger particles and eventually settled out [16]. Multiple regression technique was used to develop a response surface analysis of juice clarity as a function of enzymatic hydrolysis process variables. All the variables affected the juice clarity significantly (Table 2). The time (*X*_2_) had the most significant effect.

**Figure 3:**
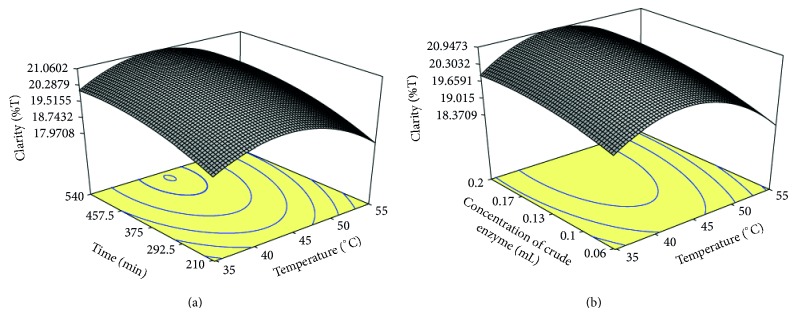
Response surface plots of clarity of juice as a function of (a) time and temperature and (b) concentration of crude pectinase enzyme and temperature.

The response surface curves were plotted to explain the interaction of the variables and to determine the optimum level of each variable (Figures 3(a) and 3(b)).  Figure 3(a) shows the effect of incubation temperature (*X*_1_) and time (*X*_2_) on juice clarity keeping the third at its middle level. It is clear from the figure that the clarity of the juice increased with both incubation time and temperature up to 509.60 min and 43.81°C temperature, respectively. Karangwa et al. [17] observed that the clarity of the blended carrot-orange juice decreased when the temperature was increased above 50°C. 

Figure 3(b) reveals the effect of incubation temperature (*X*_1_) and crude enzyme concentration (*X*_3_) on the clarity of juice. It was evident that the clarity of juice increased with increase in temperature up to 43.90°C and crude enzyme concentration of 0.19 mL/per 25 g of pulp. Degradation of the polysaccharides like pectin leads to a reduction in water holding capacity, and consequently, free water is released to the system which increases the yield and clarity of juice [18]. With further increase in the incubation temperature, the clarity of juice decreased. 

#### 3.1.3. Optimization and Verification of Process Variables

 The process was optimized by keeping the main constraints as maximum possible juice yield, maximum clarity, and minimum viscosity of juice. Under these constraints, the optimum treatment conditions were found to be incubation temperature 44.97°C, time 474.50 min, and concentration of crude enzyme 0.20 mL/25 g of pulp (Table 4). But practically, it is difficult to maintain the recommended conditions during processing, and it is expected that there may be some deviation. Hence, the optimum conditions were targeted as temperature 45°C, time 475 min, and concentration of enzyme 0.20 mL/25 g of pulp. The experiments were conducted to check the variation in juice yield, viscosity, and clarity of juice under the optimum conditions while keeping the target constraints. The results indicate a high fit degree between the observed and predicted values from the regression model. 

### 3.2. Optimization of Pretreatment Conditions Using Commercial Enzymes

The optimized conditions for the pretreatment of bael pulp using commercial enzymes were concentration of pectinase 5 mg/25 g of pulp (1.64 IU/mg), time 425 min, and temperature 47°C (Table 4), and the responses, juice recovery, viscosity, and clarity, were 84.5%, 1.35 cps, and 22.43%T, respectively, under the optimized conditions [7]. 

### 3.3. Comparative Effect of Crude and Commercial Enzymes for the Improvement of Juice Yield, Viscosity, and Clarity Using Principal Component Analysis (PCA)

 The juice yield, viscosity, and clarity from the crude enzyme treated pulp were 82.9%, 1.41 cps, and 21.32%T, respectively, under the optimized conditions. It is evident from the data (Table 5) that the crude enzyme treatment is equally competitive to the commercial enzymes. The possible cause of crude enzyme competitiveness may be a cumulative effect of other polysaccharases such as cellulases and hemicellulases along with the pectinases present in the crude enzyme.

**Figure 4:**
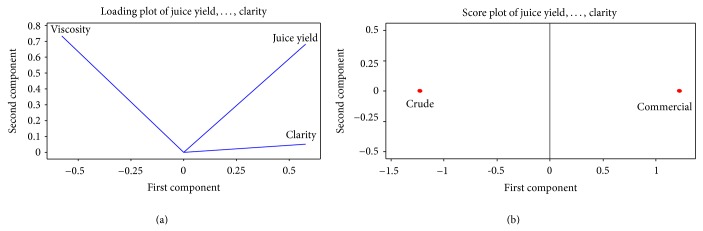
(a) Loading plot of yield, clarity, and viscosity of the juice under crude and commercial enzymes treatment. (b) Score plot of yield, clarity, and viscosity of the juice under crude and commercial enzymes treatment.

Principal component analysis (PCA) was used to obtain a lesser number of uncorrelated variables from a large set of data. The objective of principal components analysis was to explain the maximum amount of variance with the least number of principal components. The results of the analysis are shown in Figures 4(a) and 4(b). The PCA plots provide an overview of the similarities and differences between the crude and commercial enzyme treated samples and of the interrelationships among the various measured properties. The distance between the locations of both the samples on the score plot is directly proportional to the degree of difference or similarity between them (Figure 4(b)).

The score plot (Figure 4(b)) clearly indicates that the commercial enzyme treated sample shows maximum variance in first principal component, whereas crude enzyme treated sample shows maximum variance in second principal component. Therefore, the variables of commercial enzyme treated samples can be considered as significant first principal components. Maximum covariance of 57% was obtained in first principal component, whereas in second principal component, variances of 68%, 73%, and 5.1% were found for yield, viscosity, and clarity, respectively. The maximum Eigen value in the correlation matrix was 3, which was the nearest to the variance derived from clarity; thereby, the variable clarity in second principal component can be considered as a significant variable to differentiate between crude and commercial enzymes treated juices. It is evident from the loading plot of variables, that clarity and yield are correlated to higher degree as compared to the viscosity. It is suggested from this analysis that only two variables that is, clarity and yield, are to be studied for comparing samples of juice treated with either of enzymes.

## 4. Conclusions

The present study concluded that bael juice yield, viscosity, and clarity are functions of enzymatic hydrolysis conditions. Significant regression model describing the variation of juice yield, viscosity, and clarity with respect to the independent variables, temperature, time, and concentration of crude enzyme, was found adequately fit to predict the responses under study. The usage of either crude or commercial enzymes significantly enhanced juice yield and clarity as compared to the control. According to principal component analysis, it is suggested to study juice yield and clarity while comparing the different samples treated with either of the enzymes. The study also indicates the equal effectiveness and competitiveness of crude enzyme as of commercial enzymes, and thus it may be one of the important considerations to reduce the processing cost. 

## Figures and Tables

**Table 1:** Experimental range and levels of the independent variables.

Variables	Range and levels				
	−1.68	−1	0	+1	+1.68
Temp. (*X*_1_, °C)	28.18	35	45	55	61.82
Time (*X*_2_, min)	97.5	210	375	540	652.5
Conc. of crude enzyme (*X*_3_, mL)	0.01	0.06	0.13	0.20	0.25

**Table 2:** The central composite rotatable experimental design employed for enzymatic hydrolysis pretreatment of bael pulp.

Exp. no.	Coded variables			Uncoded variables			Responses		
	*X*_1_	*X*_2_	*X*_3_	Temp. (^o^C)	Time (min.)	Conc. of crude enzyme (mL)/25 g pulp	% Age yield	Viscosity (cps)	Clarity (%T)
1	−1	−1	−1	35	210	0.06	70.3	1.61	18.7
2	1	−1	−1	55	210	0.06	69.8	1.69	17.6
3	−1	1	−1	35	540	0.06	71.1	1.51	19.3
4	1	1	−1	55	540	0.06	72.8	1.49	18.5
5	−1	−1	1	35	210	0.2	77.1	1.44	18.3
6	1	−1	1	55	210	0.2	75.8	1.61	17.6
7	−1	1	1	35	540	0.2	79.6	1.42	20.5
8	1	1	1	55	540	0.2	80.1	1.45	20.1
9	−1.68	0	0	28.18	375	0.13	70.4	1.52	17.7
10	1.68	0	0	61.81	375	0.13	72.4	1.65	16.6
11	0	−1.68	0	45	97.50	0.13	73.2	1.59	18.7
12	0	1.68	0	45	652.5	0.13	80.1	1.46	20.5
13	0	0	−1.68	45	375	0.01	70.1	1.6	19.1
14	0	0	1.68	45	375	0.25	83.7	1.4	21
15	0	0	0	45	375	0.13	81.4	1.45	20.8
16	0	0	0	45	375	0.13	81.7	1.42	20.5
17	0	0	0	45	375	0.13	80.2	1.42	21.1
18	0	0	0	45	375	0.13	80.6	1.43	21
19	0	0	0	45	375	0.13	78.5	1.45	20.6
20	0	0	0	45	375	0.13	81	1.43	20.6

**Table 3:** Regression coefficients of predicted quadratic polynomial models for the responses for the model.

Coefficients	Juice yield	Viscosity	Clarity
Intercept	80.57^(a)^	1.43^(a)^	20.77^(a)^
Linear			
*A*	0.28	0.035^(a)^	−0.36^(b)^
*B*	1.63^(b)^	−0.05^(a)^	0.68^(a)^
*C*	3.77^(a)^	−0.05^(a)^	0.41^(b)^
Quadratic			
*A* ^2^	−3.26^(a)^	0.05^(a)^	−1.28^(a)^
*B* ^2^	−1.40^(b)^	0.03^(b)^	−0.41^(b)^
*C* ^2^	−1.31^(b)^	0.02^(b)^	−0.25^(b)^
Cross product			
*A*∗*B*	0.5	-0.03^(b)^	0.08
*A*∗*C*	−0.25	0.02^(b)^	0.1
*B*∗*C*	0.38	0.029^(c)^	0.4^(b)^
*R* ^2(d)^	0.9764	0.9738	0.9806
Adj. *R* ^2(e)^	0.9551	0.9503	0.9631
CV^(f)^	1.31	1.33	1.34
*F* value	45.92	41.38	56.04

Statistically significant at ^(a)^ *P* < 0.001, ^(b)^ *P* < 0.05, and ^(c)^ *P* < 0.10; ^(d)^coefficient of multiple determination; ^(e)^adjusted *R* ^2^; ^(f)^coefficient of variance.

**Table 4:** Optimization of process variables with respect to juice yield, viscosity, and juice clarity.

		Commercial enzymes			Crude enzyme	
		Optimum value (In the range)	Optimum value (Targeted)		Optimum value (In the range)	Optimum value (Targeted)

Variables	Temperature (°C)	46.57	47	Temperature (°C)	44.97	45
	Time (min)	425.21	425	Time (min)	474.50	475
	Conc. of pectinase (mg/25 g pulp)	4.96	5.00	Conc. of crude enzyme (mL/25 g pulp)	0.20	0.20

		Predicted value	Experimental value		Predicted value	Experimental value

Responses	Juice yield (%)	85.19	84.5	Juice yield (%)	83.73	82.9
	Viscosity (cps)	1.37	1.35	Viscosity (cps)	1.39	1.41
	Juice clarity (%T)	23.25	22.43	Juice clarity (%T)	21.40	21.32

**Table 5:** Comparison of crude and commercial enzymes for the improvement of juice recovery from bael.

Parameter	Control∗	Commercial enzyme treatment		Crude enzyme treatment	
		Treated	Difference	Treated	Difference
Juice yield (%)	69.1	84.5	15.40	82.9	13.8
Viscosity (cps)	1.69	1.35	0.34	1.41	0.28
Juice clarity (%T)	17.0	22.43	5.43	21.32	4.32

∗The control sample was prepared by mixing 25 g of pulp with 62.5 mL water and then filtered through cheese cloth without any enzymatic treatment.
